# Unchanged incidence and increased survival in children with neuroblastoma in Denmark 1981–2000: a population-based study

**DOI:** 10.1038/sj.bjc.6604922

**Published:** 2009-02-17

**Authors:** H Schroeder, J Wacher, H Larsson, S Rosthoej, C Rechnitzer, B L Pedersen, N L T Carlsen

**Affiliations:** 1 Department of Pediatrics, University Hospital of Aarhus, Skejby, DK 8200 Aarhus, Denmark; 2 Department of Clinical Epidemiology, University of Aarhus, DK 8000 Aarhus, Denmark; 3 Department of Pediatrics University Hospital of Aalborg, DK 9000 Aalborg, Denmark; 4 Department of Pediatrics, The Juliane Marie Center, National State Hospital of Denmark, DK 2100 København Ø, Denmark; 5 Department of Pathology, National State Hospital of Denmark, DK 2100 København Ø, Denmark; 6 Department of Pediatrics, University Hospital of Odense, DK 5000 Odense, Denmark

**Keywords:** neuroblastoma, incidence, population-based, survival, mortality

## Abstract

Treatment results for neuroblastoma in Denmark have been poorer than in other Nordic countries, so we investigated whether a change in incidence, stage distribution and survival had occurred between 1981 and 2000. Clinical data were retrieved from the medical charts of 160 children <15 years of age with extra-cranial neuroblastoma (*n*=139) or ganglioneuroblastoma (*n*=21) diagnosed in Denmark between 1981 and 2000. The minimal follow-up time was 52 months. Statistical analyses were performed in STATA. The incidence was 8.55 per million children below 15 years of age (world standard 9.6) and 42.6 per million children below 12 months of age, and it has remained unchanged since 1970. The median age at diagnosis was 27 months. In all, 32% of the children were aged below 12 months at diagnosis, 53% had metastatic disease and in 12% the diagnosis was made incidentally. Prognostic factors such as age, stage and site of primary tumour were the same as in other studies and did not change. During the study period, the mortality rate decreased steadily, and the 5-year survival rate increased from 38% in 1981–1985 to 59% in 1996–2000, corresponding to the level found in other Western countries. Increased survival was also seen in children with metastatic disease. Participation in international studies, better supportive care and possibly postoperative autologous stem cell transplantation may have contributed to the increased survival.

Neuroblastoma is the most frequent solid tumour outside the central nervous system in children below 15 years of age in Denmark with an annual incidence of 8–10 new cases. The most recent population-based study of neuroblastoma in Denmark in 1986 included 253 children diagnosed during 1943–1980 (Carlsen *et al*, 1986; Carlsen, 1994). These studies showed increases in incidence especially in children below 12 months of age, in the incidence of incidentally diagnosed neuroblastomas (Carlsen, 1990) and in the survival rate mainly because of a more favourable age and stage distribution; it was noted, however, that survival was significantly better in children who were treated with chemotherapy (Carlsen *et al*, 1986; Carlsen, 1994).

Since 1985, all Danish children with solid tumours have been registered in the Nordic Solid Tumour Registry. The annual report from the registry in 2000 showed that the survival of neuroblastoma in Denmark during 1985–1994 was significantly poorer than in the other Nordic countries, especially Finland.

We aimed to investigate whether neuroblastoma during the period 1981–2000 showed changes in incidence and mortality rate since the earlier population-based study in Denmark. We also wished to determine whether survival, especially in children with metastatic neuroblastoma, had improved and whether the distribution of known important prognostic factors (i.e. age, stage and localisation of primary tumour) had changed, both during the study period and in comparison with the earlier study (Carlsen *et al*, 1986).

## Materials and methods

Patients were identified from four different sources: (1) The National Cancer Registry of Denmark, (2) The Nordic Solid Tumour Registry, (3) the databases of the four Danish centres of paediatric oncology and (4) the histopathology databases at the Danish Departments of Pathology. A total of 160 neuroblastoma (*n*=139) or ganglioneuroblastoma (*n*=21) cases were identified in children below 15 years of age, (86 boys, 74 girls) either histologically verified (*n*=145) or with increased urinary vanillic mandelic acid (VMA) or homovanillic acid (HVA), together with the detection of malignant cells in the bone marrow (*n*=15). Minimal follow-up was 51.6 months after diagnosis, and at latest follow-up on 1st March 2007, 91 children had died, 66 were alive with no evidence of disease and three had emigrated in first complete remission, 4, 4 and 5 years, respectively, after diagnosis. The populations used were yearly averages at ages <1, 1–4 and 5–14 years during the study period as reported by Statistics Denmark (www.dst.dk). Clinical data were registered after a critical review of all medical charts. Cases of incidentally diagnosed neuroblastoma were defined as those diagnosed during a routine paediatric examination or by an examination for an unrelated disorder, such as chest X-ray, because of suspected lower pulmonary infection.

Disease stage was classified according to the International Neuroblastoma Staging System (INSS) (Brodeur *et al*, 1993, 1994):

Stage 1: Tumour localised, completely removed surgically with or without microscopic residual tumour.
Stage 2: Unilateral tumour incompletely removed, possibly with tumour in ipsilateral lymph nodes.
Stage 3: Tumour infiltrating over the midline with or without tumour tissue in regional lymph nodes; or unilateral tumour with tumour tissue in contralateral lymph nodes; or midline tumour with tumour tissue in bilateral lymph nodes.
Stage 4: Primary tumour of any stage with metastases to bone, bone marrow, remote lymph nodes, liver or other organs in children over 12 months at diagnosis.
Stage 4S: Children <12 months of age with primary tumour stage 1 or 2 and metastases to liver, skin or bone marrow infiltration (with <10% malignant cells in the bone marrow).

Treatment varied over time and by stage, that for stage 1 being primary operation. All children with stage 2 had primary operation, followed by chemotherapy in 66% and irradiation in 17% of cases. Almost all children with stages 3 and 4 were treated with chemotherapy of which 75% were treated according to international contemporary neuroblastoma protocols (OPEC/OJEC (Shafford *et al*, 1984), (Vincristine, Cisplatinum, Etoposide, Cyclophosphamide and Carboplatin; Pearson *et al*, 2008)). Irradiation of the primary tumour was given to 20% of children in stages 3 and 4.

From 1991, myeloablative chemotherapy followed by autologous stem cell reinfusion was introduced for children over 12 months of age with stage 4 disease after clearing the bone marrow of neuroblastoma cells; it consisted primarily of high-dose melphalan+/−busulfan, followed by reinfusion of autologous bone marrow cells (before 1 January 1998) or peripheral stem cells (after 1 January 1998) without purging. Operations were performed at the four university hospitals in Denmark responsible for the treatment of children with cancer. Children with stages 1 and 2 were operated at the time of diagnosis. Of the 27 children with stage 3, 20 were operated before the start of chemotherapy. Before 1990, most children with stage 4 disease were operated primarily; since 1990, however, all children over 12 months of age were operated only after 4–6 months of preoperative chemotherapy (Pearson *et al*, 2008). The extent of macroscopic radicality was stated by the operating surgeon.

The results of 23 patients with stage 4 over 12 months of age diagnosed between 1990 and 1999 were included in a multicentre international European randomised study (Pearson *et al*, 2008).

Therapy for stage 4S varied considerably.

### Statistical methods

Incidences were calculated from the background populations and age was standardised according to the world standard population, and comparisons were performed by the χ^2^ test. Survival curves were performed by the Kaplan–Meier method and compared by the log-rank test. *P*-values below 0.05 were considered statistically significant. All analyses were performed in STATA 9.

## Results

There were 86 boys and 74 girls, ratio: 1 : 1.16. The median age at diagnosis was 27 months (mean 33 months; range: 0–154 months). For children aged 12 months with metastatic disease, the median age at diagnosis was 40 months (mean 50 months; range: 12–154 months). For patients alive at the time of follow-up or at emigration (*n*=69), the mean time of follow-up was 174 months (range: 52–311 months). For those who died, the mean survival was 20 months (range: 0–189 months).

Incidental neuroblastoma was diagnosed in 12% of neuroblastoma cases (19 out of 160) (Table 1), and there was no difference in the incidence of such cases between the two 10-year periods. Fourteen cases were diagnosed in children <12 months of age, one in a 21-month-old child and four between ages 31–43 months. A tumour was discovered in the mediastinum in seven children during an X-ray examination for unrelated symptoms, and by abdominal examination in 12 children (by palpation or ultrasonography). Three of those with incidentally discovered neuroblastoma died, one with stage 3 at 3 months, 5 weeks after diagnosis, one infant with stage 4S died from a genetically inherited lung disease within 2 months of diagnosis and one tetraplegic boy with stage 3 neuroblastoma died from pneumonia unrelated to neuroblastoma 15 years after diagnosis. All seven children with incidental neuroblastoma localised in the mediastinum survived.

The incidence of all neuroblastomas during 1981–2000, by age and period, are shown in Table 2, with that in children below 15 years being 8.55 per million and 9.6 per million being age standardised. There was no difference in incidence between the two 10-year periods (*χ*^2^: *P*=0.95), either in children below or over 12 months at diagnosis (*χ*^2^: *P*=0.21 and *P*=0.30, respectively). The 52 children (including 14 incidental cases) aged below 12 months at diagnosis represented an incidence of 42.6 per million; 40% of these infants were below 3 months at diagnosis. There was no difference in the age and stage distribution between the two 10-year periods (Table 2).

**Figure 1:**
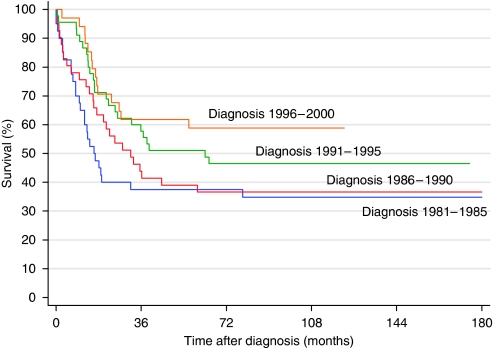
Survival of neuroblastoma in Denmark during the four 5-year periods.

**Figure 2:**
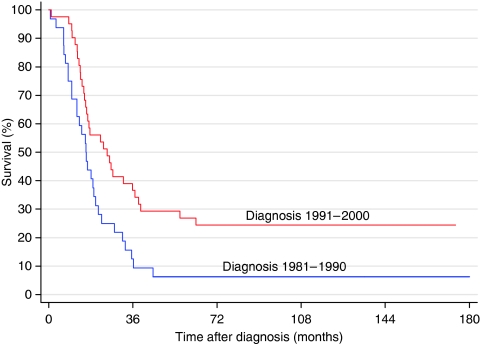
Survival of children >12 months of age with stage 4 neuroblastoma during two 10-year periods.

Figure 1 shows the survival curves for the four 5-year periods. There are significant differences between the two 10-year periods (log rank: *P*<0.05). Also, for children over 12 months of age with metastatic disease, the survival curves for the two 10-year periods differ significantly (Figure 2, log rank: *P*<0.05). For children diagnosed during 1981–1990, the overall survival (OS) was 37%, whereas it was 54% for those diagnosed during 1991–2000 (*P*=0.01) (Table 3). The OS increased from 32% (1981–1985) to 58% (1996–2000) (*P*<0.01). In univariate analysis, the localisation of the primary tumour, age at diagnosis and the stage all were significant prognostic factors (Table 3). After adjustment for stage and localisation, age had independent prognostic significance (*P*<0.001).

The survival increased significantly for children with localised tumours (stages 1–3 from 57% during 1981–1990 to 86% during 1991–2000 (*P*=0.01 Fisher's exact test).

During the first 12 months after diagnosis, the mortality rate ratio (MRR) was significantly lower during the second 10-year period when compared with the first (*P*<0.001) (Table 4). MRR was especially high for children with stages 3 and 4S during the first 6 months after diagnosis and was significantly higher than for stages 1, 2 and 4 (*P*<0.001.) A considerable contribution to the improved prognosis during the latter 10-year period was caused by a reduced mortality, especially during the first 3 months after diagnosis, as only 4% (3 out of 79) of children diagnosed during 1991–2000 died, compared with 17% (14 out of 81) of those diagnosed during 1981–1990 (*P*<0.01 Fisher's exact test) (data not shown).

Regarding the period later than 12 months after diagnosis, the MRR was lower for children diagnosed during 1991–2000 compared with 1981–90, although not statistically significant (Table 4). The MRR was significantly lowest for stages 1 and 2 more than 12 months after diagnosis, and highest for stage 4 more than 12 months after diagnosis but not earlier. For children with stages 3 and 4, no difference in OS could be shown whether the surgical procedure was radical or non-radical (data not shown).

## Discussion

In this population-based study of neuroblastoma in Denmark over a 20-year period, the age-standardised incidence was 9.6 per million overall and 42.6 per million below 12 months of age at diagnosis, similar to those found in Denmark during 1976–1980 (Carlsen, 1986, 1994) and in other population-based studies after 1975 in which no screening for neuroblastoma had taken place. The most marked increase in prognosis occurred during the past 10 years of the study period when it improved for all stages, and also for children over 12 months with metastatic disease (Bernstein *et al*, 1992; Powell *et al*, 1998; Spix *et al*, 2001, 2006; Cotterill *et al*, 2001a). Thus, no change has occurred in the incidence of neuroblastoma in Denmark during the past 30 years and, more importantly, the age distribution has not changed either.

The absence of change in incidence is in contrast to other European regions in which the overall age-standardised incidence increased from 8.4 to 11.6 during almost the same 20-year study period. This was primarily caused by an increase among 0 to 12-month-old children in all regions except the British Isles and the Nordic region (Denmark, Norway, Finland and Iceland), where the incidences were constant over the study period (Spix *et al*, 2006). The increase in neuroblastoma incidence in children below 12 months of age was closely correlated with an increase in overall survival. The increased incidence in other European regions cannot be explained by the extent of screening that has taken place only in parts of Germany during part of the study period, although an increased incidence was found in the screened population compared with the control population (Schilling *et al*, 2002). Screening was also performed in a French region and in smaller areas of Great Britain (Powell *et al*, 1998).

The increased incidence observed in some regions is probably because of an increased number of incidentally diagnosed neuroblastomas, predominantly in the infant group, especially in Germany, France and Austria. More frequent routine health checks are provided in these countries as compared with the UK and Denmark. More children are examined by paediatricians, and greater availability of ultrasound may have contributed to an increase of asymptomatic patients diagnosed incidentally. Incidental neuroblastoma was reported in 27% of Austrian and 34% of German cases during this period in contrast to 7.7% in the UK, although this covered only 15% of patients from two regions. This also explains why the number and incidence of neuroblastomas in children <12 months of age diagnosed in Denmark during our study period was lower than that in France, Germany and Austria (Powell *et al*, 1998; Spix *et al*, 2006). Incidental neuroblastomas during our study period represented 12% of cases (*n*=19), considerably lower than in Germany, France and Austria, and a little higher than the figures from two regions in the UK. There was no increased trend in incidental cases over the 20-year period and the incidence was similar to that during 1971–80 when 14% (15) of cases were detected incidentally (Carlsen, 1990).

The stage distribution in this study was unchanged during the study period and was exactly the same as during 1943–1980 in Denmark (Carlsen, 1986); 53% of patients had stage 4 disease. Compared with France, Austria and Germany, the percentage of stages 1 and 2 did not differ, about 24%, but was markedly higher than in the UK. The proportion with stage 4 was higher in Denmark, but when stage 3 was included the figures were different from those in France, Germany and Austria, but lower than those in the UK (Powell *et al*, 1998). In this material, 80% of tumours were situated in the adrenal glands or the retroperitoneum, as in the earlier study (Carlsen, 1986) and in a report from the European Neuroblastoma Study Group (Cotterill *et al*, 2000). All significant prognostic clinical parameters such as age, stage distribution and localisation of primary tumour did not differ from other international studies, in which no form of screening had taken place, or from the earlier Danish study.

This is an important observation as age below 12 months is one of the strongest prognostic factors (Carlsen, 1986; Cotterill *et al*, 2000; Spix *et al*, 2001). A large European study showed that survival was highest in Germany and France, where the incidence below 12 months was high, about 60 per million, whereas that in England was 50% lower (Powell *et al*, 1998). In Finland, which has the highest survival in the Nordic countries, a little more than 50% of children with newly diagnosed neuroblastoma were below 12 months compared with only 33% of Danish children (Spix *et al*, 2001).

We showed a significant decrease in the MRR during the study period, implicating that survival has increased significantly because of improved therapy. Corresponding to this, a significantly better survival was shown for children diagnosed during 1991–2000 compared with 1981–1990.

The observed increase in the 5-year survival of neuroblastoma in Denmark during the four 5-year periods from 38% during 1981–85 to 59% during 1996–2000 is not explained by a change in age or stage distribution, which was unchanged during the period and also unchanged compared with the preceding 10-year periods (Carlsen *et al*, 1986). Survival in Denmark, Norway and Finland during 1993–97 was 61% (Spix *et al*, 2006), so the observed survival rate of 59% in Danish children during 1996–2000 is probably not significantly different from that of the two other Nordic countries, and as the minimum follow-up is about 5 years, the figures are not expected to change significantly (Cotterill *et al*, 2001b).

However, the survival rates in other Western countries, mainly France and Germany, during 1993–1997 was 74%, probably significantly higher than in Denmark during this period. One explanation for this difference may be that the age-standardised neuroblastoma rate was about 30% higher in the Western countries than in Denmark during this period, almost exclusively explained by a much higher incidence in children below 12 months of age (Spix *et al*, 2006). This fact may render comparisons of overall survival between the two groups difficult. It seems, however, that the survival at 2–14 years was still higher in the Western than in Nordic countries (53 *vs* 42%) (Spix *et al*, 2006).

A considerable part of the improved prognosis from the first to the second 10-year period could be attributed to a significantly decreased early mortality, especially during the first 6 months after diagnosis during the second period. The survival of children with stages 3 and 4S was considerably lower compared with other studies, especially for early deaths. For stage 3, this was caused by postoperative complications during the first 10-year period after attempts at radical excision without neoadjuvant chemotherapy in children with large abdominal tumours. During the second period, most patients with stage 3 had preoperative chemotherapy allowing the tumour to shrink and rendering secondary operation less risky.

Our analyses also showed a significantly increased survival of children over 12 months with stage 4 neuroblastoma treated after 1990, in which autologous stem cell transplantation (ASCT) was introduced compared with the earlier period. As initial response to rapid COJEC was superior to OPEC/OJEC in a randomised study of initial chemotherapy (Pearson *et al*, 2008), chemotherapy may, in addition to myeloablative therapy, have had an impact on the increased survival in this group treated after 1990. Also, Berthold *et al* (2003) showed an increased survival in children over 12 months of age with stage 4 disease between 1990 and 2001, with a survival of 44% during 1997–2001.

Multivariate analyses showed that the well-known prognostic factors of age and stage, but not localisation of the primary tumour, had independent prognostic significance, as had the time of diagnosis. Myeloablative chemotherapy and ASCT had prognostic significance in univariate analysis, but they had no independent prognostic significance in multivariate analyses. The effect of the completeness of the operation in children with stages 3 and 4 had prognostic significance neither in uni- nor multivariate analyses.

In a population-based study of all cases of neuroblastoma in Denmark during 1981–2000, no increase in incidence and no change in the distribution of prognostic factors were shown compared with a study up to 1980. The most prominent increase in prognosis occurred during the second 10 years of the study period when this improved for all stages, and also for children over 12 months with metastatic disease.

## Figures and Tables

**Table 1:** The age and stage distribution, and the localisation of the primary tumour in incidental cases

	**Total *N***	**Age 0–11 months**	**Age 12–23 months**	**Age 24+ months**
*Stage*				
1	6	3	1	2
2	4	2		2
3	7	7		
4S	2	2		
Total	19	14	1	4
Localisation of primary tumour				
Mediastinum	7	5		2
Retroperitoneum/adrenal glands	10	8	1	1
Pelvis	2	1		1

**Table 2:** Age-specific incidence and stage distribution of neuroblastoma and ganglioneuroblastoma

	**1981–1990 *N***	**1991–2000 *N***	**Incidence per million during the study period**
*Age at diagnosis*			
<12 months	28	24	42.6
12–23 months	9	13	16.1
24–59 months	28	30	
>59 months	16	12	2.2
Total	81	79	
Age-specific incidence per million	8.51	8.60	8.55
Age-standardised incidence			9.6
			
*Stage at diagnosis*			
Stage 1	4	8	7.5%
Stage 2	15	11	16.3%
Stage 3	18	9	16.9%
Stage 4	38	46	51.3%
Stage 4S	6	5	7.0%

**Table 3:** Number of patients, number of deaths, 5-year survival (%) with 95% CI

	**Number of children**	**Number of deaths**	**5-year survival (95 % CI)**	**Log-rank test for similar pattern of survival (*P*)**
*Period of diagnosis*				
1981–1990	81	53	37 (27–47)	*P*=0.01
1991–2000	79	38	54 (43–65)	
				
*Age (months)*				
<12	52	14	77 (63–86)	*P*<0.001
12–23	22	11	50 (28–68)	
24–59	58	41	31 (20–43)	
⩾60	28	25	14 (5–30)	
				
*Stage*				
1, 2	38	3	92 (77–97)	*P*<0.001
3	27	17	44 (26–62)	
4	84	66	23 (14–32)	
4S	11	5	64 (30–85)	
				
*Localisation*				
Neck/mediastinum/pelvis	31	7	77 (58–88)	*P*<0.001
Abdominal/adrenal	129	84	38 (30–46)	

CI=confidence interval.

Log-rank test for similar patterns of survival.

**Table 4:** MRR for each of the factors: diagnosis period, age, stage and localisation of the primary tumour divided into the periods 0–12 months and >12 months after diagnosis

	**Adjusted MRR (95% CI)**	
	**0–12 months after diagnosis**	**>12 months after diagnosis**
*Diagnosis period*		
1981–1990	1	1
1991–2000	0.30 (0.13–0.66)	0.62 (0.36–1.06)
		
*Age (months)*		
<12	1	1
12–23	1.42 (0.45–4.45)	2.69 (0.78–9.29)
24–59	0.83 (0.31–2.24)	5.60 (1.99–15.74)
⩾60	1.78 (0.65–4.86)	5.80 (1.96–17.17)
		
*Stage*		
1, 2	0.47 (0.05–4.33)^a^	0.03 (0.00–0.26)
	0.15 (0.02–1.23)^b^	
3, 4S	6.15 (2.04–18.57)^a^	0.53 (0.24–1.15)
	0.21 (0.03–1.63)^b^	
4	1	1
		
*Localisation*		
Neck/mediastinum/pelvis	1	1
Abdominal/adrenal	1.09 (0.36–3.25)	1.77 (0.52–6.00)

CI=confidence interval; MRR=mortality rate ratio.

a During the period 0–6 months after diagnosis.

b During the period 6–12 months after diagnosis.
