# Unchanged incidence and increased survival in children with neuroblastoma in Denmark 1981–2000: a population-based study

**DOI:** 10.1038/sj.bjc.6605006

**Published:** 2009-03-31

**Authors:** H Schroeder, J Wacher, H Larsson, S Rosthoej, C Rechnitzer, B L Petersen, N L T Carlsen

**Correction to**: British Journal of Cancer (2009) **100,** 853–857. doi: 10.1038/sj.bjc.6604922

During submission of the above article to the journal, one of the authors' surnames was incorrectly spelt - BL Petersen's name was published as ‘BL Pedersen’. The authors are now happy to correct this mistake.

